# Current Perspectives on Proteus Species: From Morphology to Multidrug Resistance

**DOI:** 10.7759/cureus.115116

**Published:** 2026-08-24

**Authors:** Prital B Kulkarni, Satyajeet K Pawar, Satish R Patil

**Affiliations:** 1 Department of Microbiology, Krishna Institute of Medical Sciences, Krishna Vishwa Vidyapeeth (Deemed to be University), Karad, IND

**Keywords:** biofilm formation, multidrug-resistant, proteus species, swarming, virulence factors

## Abstract

*Proteus* species are Gram-negative, highly motile opportunistic pathogens belonging to the family Enterobacteriaceae and are widely distributed in the environment, including the human digestive tract and animals. Among them, *Proteus mirabilis* is the most clinically significant species and is commonly associated with catheter-associated urinary tract infections (CAUTIs), wound infections, gastrointestinal disorders, and other nosocomial infections. The pathogenicity of *Proteus* is attributed to several virulence factors, including swarming motility, urease production, fimbriae, adhesins, hemolysin, lecithinase, and biofilm formation, which facilitate colonization, tissue invasion, immune evasion, and persistence within the host. The genus also possesses complex antigenic structures involving O, H, and K antigens that contribute to serological diversity and pathogenic variability. Increasing antimicrobial resistance among *Proteus* isolates, particularly through the production of extended-spectrum β-lactamases (ESBLs), AmpC β-lactamases, and carbapenemases, has become a major clinical concern. These resistance mechanisms significantly limit therapeutic options and complicate infection control measures. This review highlights the classification, morphology, virulence factors, pathogenicity, clinical manifestations, antimicrobial resistance mechanisms, and phenotypic characteristics of *Proteus* species, emphasizing their growing importance as multidrug-resistant (MDR) opportunistic pathogens.

## Introduction and background

The genus *Proteus* belongs to the family Enterobacteriaceae and consists of Gram-negative, highly motile bacteria that are widely distributed in soil, water, sewage, and the intestinal tracts of humans and animals. Among these species, *Proteus mirabilis* is the most clinically significant and is frequently associated with urinary tract infections (UTIs), particularly catheter-associated urinary tract infections (CAUTIs) [1]. *Proteus* species are distinguished by their exceptional swarming motility, urease production, and characteristic bull’s-eye colony appearance on solid media. Morphologically, they are rod-shaped, non-spore-forming bacteria possessing peritrichous flagella that facilitate movement across surfaces [1]. The antigenic structure of *Proteus* includes O (somatic), H (flagellar), and occasionally K (capsular) antigens, which contribute to serological classification and pathogenic variability among strains. These antigens play a significant role in host interaction, immune evasion, and epidemiological identification [2]. The pathogenicity of *Proteus* species is mediated by numerous virulence factors, including fimbriae, adhesins, urease, hemolysin, proteases, biofilm formation, and swarming behavior. These factors facilitate adhesion to epithelial surfaces, colonization of urinary catheters, tissue invasion, stone formation, and persistence within the host [1]. Clinically, *Proteus* species are associated with various infections, including UTIs, wound and burn infections, bacteremia, gastrointestinal infections, and, occasionally, infective endocarditis. Their strong ability to form biofilms on indwelling medical devices significantly contributes to chronic and recurrent infections [1]. Growing antibiotic resistance among *Proteus* isolates has become a major concern in recent years. Resistance mechanisms, such as the production of extended-spectrum β-lactamases (ESBLs), AmpC β-lactamases, and carbapenemases, have complicated treatment strategies and infection control practices [2]. Therefore, understanding the classification, morphology, antigenic structure, virulence mechanisms, pathogenicity, clinical manifestations, and resistance phenotypes of *Proteus* species is necessary for precise diagnosis, successful treatment, and the development of preventive strategies against these clinically important opportunistic pathogens [1].

## Review

Methodology

Search Strategy

**Figure 1:**
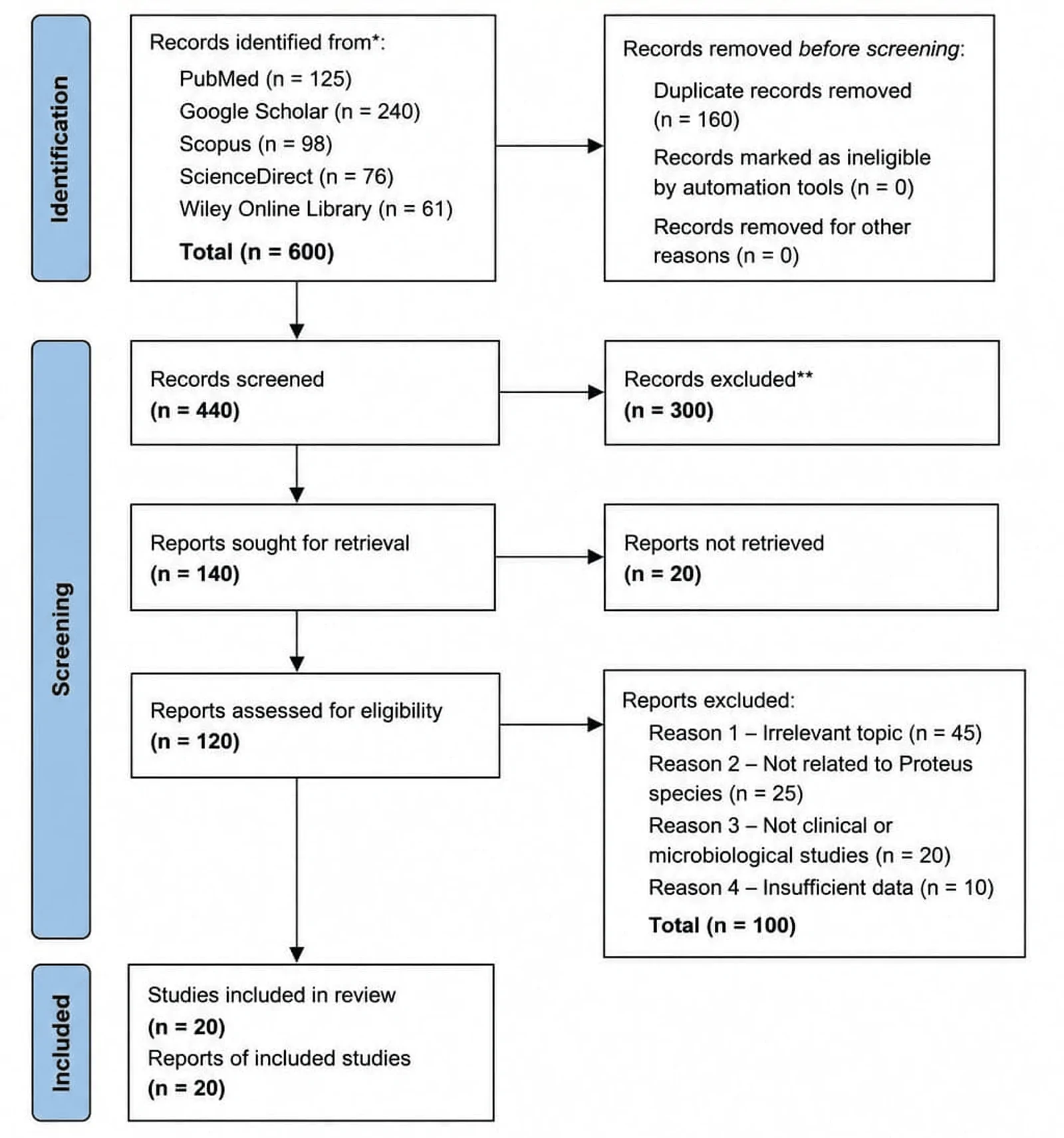
Search strategy n, number of studies *Records identified from each database searched are reported separately. **Records excluded during the screening process based on the predefined inclusion and exclusion criteria.

A narrative literature review was conducted to summarize current evidence on the morphology, virulence factors, pathogenicity, biofilm formation, clinical manifestations, and antimicrobial resistance of *Proteus* species. Electronic databases, including PubMed, Google Scholar, Scopus, ScienceDirect, and Wiley Online Library, were searched for studies published between 2000 and 2026 using relevant keywords related to *Proteus* species and antimicrobial resistance, including *Proteus* species, *Proteus* *mirabilis*, swarming motility, biofilm formation, virulence factors, antimicrobial resistance, and multidrug resistance (MDR). A total of 600 records were identified. After removing 160 duplicate records, 440 records were screened, of which 300 were excluded. A total of 140 full-text articles were sought for retrieval, and 120 were assessed for eligibility after excluding 20 unavailable reports. Following full-text assessment, 100 articles were excluded due to irrelevance, lack of focus on *Proteus* species, insufficient microbiological or clinical data, or failure to meet the inclusion criteria. Finally, 20 studies were included in the qualitative review. The selected studies were critically reviewed and synthesized to provide an overview of the classification, morphology, virulence factors, pathogenicity, clinical significance, and antimicrobial resistance mechanisms of *Proteus* species. A PRISMA flowchart of the methodology used for this review is shown in Figure 1.

History

The ability of microorganisms belonging to the genus *Proteus* to swarm on solid surfaces was first reported by the German microbiologist Gustav Hauser in 1885. The genus *Proteus* derives its name from the Greek sea god *Proteus*, who was believed to have the ability to change his shape and undergo endless transformations. This name is thought to reflect the remarkable swarming motility of the organism, which is enabled by its peritrichous flagella. The characteristic "bull's-eye" colony pattern may also have contributed to the choice of the name [3-6]. The species name *P. mirabilis* may have originated from Latin words meaning "amazing," "marvelous," and "splendid." Hauser may have named *Proteus vulgaris* from a Latin term meaning "widespread" or "ordinary," as he considered it to be the more common and typical species [5].

Classification and nomenclature

Hauser described two species of the genus, *P. vulgaris* and *P. mirabilis*, based on their ability to ferment maltose. *P. vulgaris* strains were subsequently divided into three biogroups according to three biochemical reactions: esculin hydrolysis, salicin fermentation, and indole production. Biogroup 1 was identified as *Proteus penneri*, which was negative for all three reactions. In contrast, Biogroup 2 retained the name *P. vulgaris* and was positive for all three reactions. Biogroup 3 was positive for indole production but negative for esculin hydrolysis and salicin fermentation and was therefore named *Proteus hauseri*. However, genospecies 4, 5, and 6 remained unnamed because of their similar phenotypic characteristics. Furthermore, based on phylogenetic, morphological, chemotaxonomic, and genotypic investigations, six newly described species, *Proteus terrae*, *Proteus cibarius*, *Proteus alimentorum*, *Proteus columbae*, *Proteus faecis*, and *Proteus cibi*, were recently proposed. Thus, there are currently 10 officially recognized *Proteus* species and three unidentified genospecies. The classification of additional *Proteus* species and genospecies, except for these six recently identified species, was based on variations in DNA-DNA hybridization and biochemical reactions established at least 19 years ago. More recently, the boundaries separating closely related bacterial species, subspecies, and strains have been more accurately defined using multilocus sequence analysis (MLSA) based on several housekeeping genes (HKGs) [4,7]. *Proteus* bacteria can also be distinguished by the diversity of their O antigens. To date, 80 O-antigen serogroups have been identified within the genus, some of which are further subdivided, and numerous new O serotypes continue to be discovered [5].

Morphology and cultural characteristics

*Proteus* bacteria are extremely polymorphic. They are Gram-negative rods measuring 0.4-0.6×1-3 µm, are motile (possessing peritrichous flagella), do not form spores, and do not possess a characteristic capsule. A thin, 2-3 nm peptidoglycan layer is interconnected with the wavy outer membrane. Beneath the periplasm lies a thin cytoplasmic membrane. Although *Proteus* proteins do not form traditional capsules, a fibrous capsular polysaccharide with fibers perpendicular to the cell surface has been identified in *P. mirabilis* [8].

Culture

*Proteus *spp. are easy to culture because they can grow on basic nutritional media (pH 7.2-7.4) [8]. *Proteus* grows as swarming colonies on blood agar and as non-lactose-fermenting colonies on MacConkey agar. *Proteus* exhibits urea hydrolysis, citrate utilization, H₂S production with gas formation on Triple-Sugar-Iron (TSI) agar, and a positive phenylalanine deaminase (PPA) test. The indole test distinguishes *P. vulgaris* (indole-positive) from *P. mirabilis* and *P. penneri* (indole-negative). *P. mirabilis* and *P. penneri* can be further differentiated using the maltose fermentation test [9-11]. Temperatures between 10°C and 43°C are suitable for the growth of *Proteus*. However, a temperature of approximately 37°C is optimal for strains that are part of the normal flora of humans and animals [8]. *Proteus* possesses peritrichous flagella, which are essential for motility. These flagella enable the bacteria to move across solid surfaces, producing a characteristic swarming pattern. When swarmer cells are suspended in a liquid medium, they differentiate into vegetative cells [3,8]. During this process, the original colony undergoes cell differentiation. The multinucleated swarmer cells range from 20-30 µm to 80-100 µm in length and possess 50-500 times more flagella than the vegetative cells from which they develop. The swarming process involves three phases. The first phase is differentiation, during which vegetative cells transform into elongated, hyperflagellated swarmer cells. The consolidation phase occurs when these swarmer cells rapidly move away from the colony over the surface of solid media. As their movement gradually ceases, the swarmer cells differentiate back into vegetative cells [8]. These three stages are repeated, resulting in the formation of concentric rings on the surface of the medium and producing the characteristic swarming appearance of the colonies. Different *Proteus* species can also be distinguished by their swarming behavior on the same agar plate. When two identical isolates are streaked far apart on an agar plate, the swarming edges of their colonies merge. In contrast, when two different isolates are streaked, a visible demarcation line forms where the swarming fronts meet. This line, which separates the swarming colonies of the two isolates, is called the Dienes line, and the phenomenon is known as the Dienes phenomenon [12].

Virulence factors

O, H, and K Antigens

*Proteus* bacteria possess a complex antigenic structure that includes O, H, and, in certain strains, K antigens. O antigens are somatic antigens composed of lipopolysaccharides (LPS). These O antigens are used to determine the serogroups of clinical *Proteus* strains. Approximately 20 H antigens and 50 O antigens have been identified in *Proteus* [8]. Limited research has been conducted on the H (flagellar) and K (capsular) antigens.

Fimbriae and Adhesins

The pathogenesis of *Proteus* infections depends on adhesion to epithelial surfaces. This process is initiated when molecules on the surface of the pathogen interact with molecules on host cells, resulting in physicochemical interactions that facilitate bacterial attachment. Pathogens capable of adhering to host cells produce adhesins, which are protein or glycoprotein molecules present on the surface of microbial cells. In members of the family Enterobacteriaceae, including Proteus, adhesins are primarily located on fimbriae. Members of the genus *Proteus* possess a variety of fimbriae that differ mainly in their structure. Thick fimbriae, measuring approximately 7 nm in diameter, are known as MR/P (mannose-resistant *Proteus*-like) fimbriae, whereas thin fimbriae, measuring approximately 4 nm, are referred to as MR/K (mannose-resistant *Klebsiella*-like) fimbriae. MR/P fimbriae have been shown to promote bacterial adherence to epithelial cells more effectively than MR/K fimbriae [2,8,13].

Urease

Urease is a cytoplasmic, nickel-dependent metalloenzyme that plays an essential role in the pathogenesis of *Proteus* infections. It increases the pH of urine by hydrolyzing urea into ammonia and carbon dioxide. As the urinary pH rises, calcium and magnesium phosphates precipitate from solution, leading to the formation of struvite (magnesium ammonium phosphate) and apatite (calcium phosphate) crystals. This process results in the development of urinary stones, including struvite and calcium phosphate (apatite) calculi. The effects also extend to urinary catheters, where bacteria and precipitated minerals form crystalline biofilms that progressively obstruct the flow of urine. In addition, urease-mediated alkalinization of the local environment enables *Proteus* to survive and proliferate under otherwise unfavorable conditions [2,3,13,14].

Lecithinase

Lecithinase is widely recognized as a virulence factor that enhances the pathogenicity of numerous bacteria. *P. vulgaris* and *P. mirabilis* strains exhibiting strong lecithinase activity are frequently isolated from patients with purulent inflammatory infections. The enzyme is produced more abundantly by the O-form of these strains, particularly following their transition from the H-form to the O-form [8].

Hemolysin

Hemolysin is a potent cytotoxin that damages the membranes of host cells and serves as an important virulence factor. The genus *Proteus* produces two distinct cytotoxic hemolysins, HpmA and HlyA. *P. mirabilis* and the majority of *P. vulgaris* strains produce only HpmA, whereas most *P. penneri* strains produce HlyA. A few *P. vulgaris* strains produce both HpmA and HlyA. The production of these hemolysins is closely associated with the swimming-swarming cycle of the bacterial cells. Swarming cells exhibit approximately 18-fold greater cytotoxicity than swimmer cells. Furthermore, HpmA has been shown to lyse erythrocytes, bladder epithelial cells, B-cell lymphoma cells, and neutrophils. Hemolysin facilitates bacterial invasion of host tissues and the establishment of infection. Lysis of red blood cells releases essential nutrients that promote bacterial growth within the host. Additionally, hemolysin contributes to the severity and recurrence of infections by disrupting host cell membranes, thereby facilitating bacterial proliferation and evasion of the host immune response [3,13,14].

Biofilm Production

Biofilm formation is a crucial multistage virulence mechanism that protects *Proteus* from host immune responses and antimicrobial therapy [15]. The composition of *Proteus* biofilms is highly dependent on the growth medium. In laboratory broth, they form multilayered structures, whereas in urine they develop distinctive, flat crystalline biofilms [16]. The formation of these crystalline biofilms begins when bacteria adhere to biotic or abiotic surfaces, such as silicone or latex polymers used in long-term urinary catheters [15,16]. This virulence trait plays a central role in the development of CAUTIs by promoting catheter encrustation and blockage [16]. As a result, patients may develop urinary retention and painful bladder distension, which can rapidly progress to vesicoureteral reflux, pyelonephritis, or life-threatening urosepsis [15]. The pathogenicity of *Proteus* biofilms is further enhanced by the activity of the structurally encoded urease enzyme [16]. Urease hydrolyzes urea naturally present in urine into ammonia and carbon dioxide, resulting in a marked increase in urinary pH [15,16]. This alkaline environment promotes the precipitation of calcium phosphate (apatite) and magnesium ammonium phosphate (struvite) crystals [16]. These mineral aggregates become deposited on the catheter surface and are embedded within the self-produced exopolysaccharide matrix together with bacterial cells [16].

Table 1 provides a brief summary of the major virulence factors of *Proteus* species.

**Table 1:** Virulence factors summary CAUTIs, catheter-associated urinary tract infections

Category	Factor	Function	Clinical significance
Virulence factor	Swarming motility	Mediated by peritrichous flagella; enables migration across solid surfaces and colonization of host tissues	Promotes catheter colonization, biofilm formation, and dissemination of infection
	O, H, and K antigens	Antigenic structures involved in serological diversity and immune interaction	Contribute to immune evasion and pathogenic variability
	Fimbriae and adhesins (MR/P, MR/K)	Facilitate attachment to epithelial cells and urinary tract surfaces	Essential for colonization and initiation of infection
	Urease	Hydrolyzes urea into ammonia, increasing urine pH	Causes urinary stone formation, catheter encrustation, and CAUTIs
	Lecithinase	Membrane-damaging enzyme associated with increased pathogenicity	Enhances tissue damage and inflammatory responses
	Hemolysin (HpmA, HlyA)	Cytotoxic toxin causing lysis of erythrocytes and host cells	Facilitates tissue invasion, nutrient acquisition, and persistence
	Biofilm formation	Produces protective extracellular matrix on biotic and abiotic surfaces	Promotes chronic infection, antibiotic resistance, and catheter blockage

Clinical manifestations

Species of the genus *Proteus* are commonly found in humans, animals, and the environment. They can be readily isolated from sewage, soil, garden vegetables, and various other environmental sources. *P. mirabilis* is the most prevalent species in humans and accounts for approximately 90% of all infections caused by *Proteus*spp.* P. mirabilis* is also frequently isolated from immunocompromised individuals, particularly those with infected burn wounds, and is commonly associated with the intestinal flora of humans and animals [8,17,18]. Researchers commonly isolate *P. mirabilis* and *P. vulgaris* from human clinical specimens [17]. These bacteria can cause a wide range of infections, including CAUTIs, wound and soft tissue infections, gastrointestinal infections, infectious gastroenteritis, and various hospital-acquired infections.

CAUTIs

*Proteus* species are frequently associated with UTIs in individuals with long-term urethral catheterization. Their ability to form biofilms plays a crucial role in the establishment and persistence of these infections. Biofilms protect bacteria from the host immune response, reduce the penetration of antibiotics and antibodies, and facilitate nutrient availability. Colonization of urinary catheters and chronic wounds by biofilm-producing *Proteus* species often leads to treatment failure because of increased antimicrobial resistance [19,20]. Biofilms produced by *P. mirabilis* on urinary catheter surfaces have been extensively investigated. During *P. mirabilis*-associated UTIs, the bacterium contributes to the formation of up to 30% of all urinary tract stones. In CAUTIs, crystalline biofilms are particularly problematic because biofilm encrustation leads to catheter blockage [21]. *P. mirabilis* and *P. vulgaris* are the principal *Proteus* species implicated in CAUTIs because of their ability to produce biofilms [19,20].

Tissue and Wound Infections

*Proteus* species are common causes of wound infections, particularly among hospitalized, immunocompromised, and burn wound patients. They are frequently isolated from traumatic wounds, postoperative wounds, diabetic ulcers, burns, cellulitis, abscesses, osteomyelitis, and other pathological wounds [18]. *P. mirabilis* and *P. vulgaris* are among the most common causative species associated with these infections [17,18]. Tissue invasion is facilitated by the cytotoxin hemolysin, which damages host cells and promotes bacterial survival and multiplication within host tissues [3,13,14]. A study published in 2021 reported that *Proteus *spp. was the second most common cause of wound infections after *Pseudomonas aeruginosa*. Although the study primarily focused on burn wound infections, it also highlighted that these infections are predominantly hospital-acquired and are difficult to treat because of the increasing prevalence of MDR isolates [17,18].

Gastrointestinal Infections

*Proteus* species are considered opportunistic pathogens of the gastrointestinal tract and are normally present as part of the intestinal flora in humans. However, under conditions such as immunosuppression, intestinal inflammation, or disruption of the normal gut microbiota, they may contribute to gastrointestinal diseases. *P. mirabilis* has been associated with infectious gastroenteritis, inflammatory bowel diseases such as Crohn’s disease and ulcerative colitis, and small intestinal bacterial overgrowth. Its pathogenicity is attributed to virulence factors, including urease, LPS, flagella, hemolysin, and biofilm formation, which stimulate inflammatory responses and promote bacterial survival within the gastrointestinal tract. Ammonia, produced by bacterial urease through the hydrolysis of urea, serves as a source of nitrogen for respiration and amino acid synthesis. Because *Proteus* species are Gram-negative bacteria that produce immunostimulatory flagellin proteins and LPS, growing evidence suggests that they may directly contribute to the pathogenesis of inflammatory bowel disease. Furthermore, their MDR and ability to persist within the gastrointestinal tract make them important opportunistic pathogens in hospital-acquired and chronic intestinal infections [13].

Infective Endocarditis

Infective endocarditis caused by *Proteus* species is extremely rare. A study published in 2020 reported that the condition was most commonly observed in patients with UTIs. The study also suggested that the primary contributing factor was the ability of *Proteus* species to form biofilms. Commonly affected sites included the aortic valve, mitral valve, tricuspid valve, mural endocardium, and Eustachian valve. Clinical manifestations included persistent fever, sepsis, and, in rare cases, heart failure. Although the infection is uncommon and standardized treatment guidelines are lacking, most reported cases have been treated with aminoglycosides, cephalosporins, and aminopenicillins [22,23].

Table 2 provides a brief summary of the clinical manifestations caused by *Proteus* species.

**Table 2:** Clinical manifestations summary CAUTI, catheter-associated urinary tract infections; UTI, urinary tract infections

Category	Factor	Characteristics	Clinical significance
Clinical manifestation	CAUTIs	Commonly caused by *Proteus mirabilis* and *Proteus vulgaris* through biofilm formation	Leads to recurrent UTIs, catheter obstruction, pyelonephritis, and urosepsis
	Wound and tissue infections	Frequently isolated from burns, ulcers, abscesses, cellulitis, and post-operative wounds	Associated with hospital-acquired infections and multidrug resistance
	Gastrointestinal infections	Opportunistic involvement in gastroenteritis and inflammatory bowel disease	Contributes to intestinal inflammation and chronic gastrointestinal disorders
	Infective endocarditis	Rare complication often associated with preceding UTI	May result in persistent fever, sepsis, and heart valve involvement

Laboratory diagnosis of *Proteus* spp.

Phenotypic Characters

ESBL production: Antibiotic resistance among Gram-negative bacilli is an evolving challenge due to the organisms' potential for mutation and their ability to acquire and transmit plasmids and other genetic elements that encode resistance genes. β-lactam antibiotics are the most commonly prescribed antimicrobial agents worldwide, and more than 340 distinct β-lactamases have been identified, including ESBLs, AmpC β-lactamases, and carbapenemases. ESBLs are a specific group of β-lactamase enzymes produced by Gram-negative bacteria of the family Enterobacteriaceae that hydrolyze β-lactam antibiotics. The principal ESBL-producing organisms include *Escherichia coli*, *Klebsiella pneumoniae*, and *Proteus* spp. ESBLs remain a significant clinical concern because they are plasmid-mediated and can be readily transmitted between bacterial species. The major characteristics of ESBL-mediated resistance in Enterobacteriaceae include resistance to aminopenicillins, carboxypenicillins, second-generation cephalosporins, and one or more third- and fourth-generation cephalosporins, as well as aztreonam. Bacterial isolates resistant to indicator antibiotics such as ceftriaxone, ceftazidime, or cefotaxime are suspected to be ESBL producers. Several methods, including the double-disk synergy test, ESBL E-tests, the combination disk method, and automated systems such as VITEK 2, are used for the detection of ESBL-producing organisms [24-26]. Research articles published in 2016 and 2022 reported that 54% and 34.5%, respectively, of the *Proteus* isolates obtained from clinical specimens were ESBL producers [27,28].

AmpC β-lactamase production: AmpC β-lactamases are enzymes produced by members of the family Enterobacteriaceae, including *Proteus*, that confer resistance to several β-lactam antibiotics. There is currently no standardized phenotypic test for the identification and confirmation of AmpC β-lactamase-producing Enterobacteriaceae, as numerous detection methods have been described [29]. This presents a significant challenge in the appropriate management of bacterial infections. One commonly used screening method for AmpC β-lactamase production is based on cefoxitin susceptibility testing [30]. Another approach is the inhibitor-based method, in which inhibitors such as cloxacillin and boronic acid are used; among these, cloxacillin has been shown to be the more effective inhibitor [30]. Other methods, including E-tests and polymerase chain reaction (PCR), are also employed for the detection of AmpC-producing isolates. PCR is considered the gold standard because it confirms the presence of plasmid-mediated AmpC genes, including those identified in cefoxitin-resistant *P. mirabilis* isolates [29,30].

Carbapenemase production: The increasing incidence of carbapenem-resistant *Proteus*, particularly *P. mirabilis*, has become a major threat to effective infection control. *Proteus* exhibits multifactorial resistance to carbapenems, which is primarily mediated by the horizontal acquisition of carbapenemase enzymes belonging to Ambler classes A, B, and D. In some cases, resistance is also driven by structural alterations such as porin loss, efflux pump overexpression, and modifications of penicillin-binding proteins [31].

Class A carbapenemases are rare in *Proteus* species. The first case involving a carbapenemase-producing *P. mirabilis* strain was reported in the United States in 2008. A subsequent clinical case was identified in China, where the carbapenemase-producing strain was isolated from a patient with a bloodstream infection. Antimicrobial susceptibility profiles indicate that these strains exhibit extensive MDR. They are highly resistant to carbapenems (imipenem and meropenem), broad-spectrum cephalosporins (cefazolin and cefotaxime), penicillins, and several β-lactam/β-lactamase inhibitor combinations (amoxicillin-clavulanic acid and ampicillin-sulbactam). In addition, resistance extends to multiple non-β-lactam antimicrobial classes, including fluoroquinolones (ciprofloxacin, levofloxacin, and moxifloxacin), aminoglycosides (gentamicin), phenicols, tetracyclines, and folate pathway inhibitors (sulfamethoxazole-trimethoprim). Despite this extensive resistance profile, these MDR strains have remained susceptible to a limited number of therapeutic agents, including ceftazidime, amikacin, aztreonam, and piperacillin-tazobactam [32].

Class B carbapenemases, also known as metallo-β-lactamases (MBLs), are zinc-dependent enzymes capable of hydrolyzing almost all β-lactam antibiotics, including penicillins, extended-spectrum cephalosporins, and carbapenems [32].* Proteus* species, particularly *P. mirabilis*, are important opportunistic pathogens frequently isolated from both community-acquired and nosocomial infections, especially UTIs [31]. Historically,* Proteus* species lacked chromosomally encoded β-lactamases and were therefore naturally susceptible to β-lactam antibiotics. However, they have rapidly acquired resistance through plasmid-mediated gene transfer [31]. The acquisition of carbapenemases, particularly Class B MBLs, is a major mechanism responsible for resistance to last-line carbapenem antibiotics [32]. MBLs efficiently hydrolyze a broad range of β-lactam antibiotics, including carbapenems, cephalosporins, and penicillins [33]. Phenotypically, these enzymes require zinc ions at their active sites for catalytic activity. This characteristic is utilized in laboratory detection methods, where metal chelators such as ethylenediaminetetraacetic acid (EDTA) inhibit MBL activity in disk synergy tests [33]. Furthermore, MBL-producing *Proteus *isolates frequently harbor additional resistance mechanisms, including plasmid-mediated AmpC β-lactamases and ESBLs. Combined with their intrinsic resistance to colistin, tetracycline, and nitrofurantoin, these characteristics pose a significant public health concern by leaving clinicians with very limited therapeutic options [31-33].

*Proteus* species are generally highly susceptible to carbapenems. However, they can acquire Class D carbapenemase enzymes through horizontal gene transfer from other bacterial species. Unlike Class B carbapenemases, Class D enzymes are characterized by poor inhibition with conventional laboratory inhibitors such as EDTA or clavulanic acid. A major diagnostic challenge associated with Class D-producing *P. mirabilis* is that these enzymes often exhibit relatively weak carbapenem-hydrolyzing activity. Consequently, affected strains may appear susceptible during routine laboratory susceptibility testing with carbapenems such as imipenem and meropenem, as well as with β-lactam/β-lactamase inhibitor combinations such as piperacillin-tazobactam. In addition, these Class D-producing strains frequently acquire resistance to several other first-line antimicrobial agents. Co-resistance to aminoglycosides, sulfonamides, trimethoprim, chloramphenicol, and fluoroquinolones has been reported, resulting in extensively drug-resistant (XDR) phenotypes that leave clinicians with very limited therapeutic options [31,32].

Table 3 provides a brief summary of the resistance mechanisms of *Proteus* species against different classes of antibiotics.

**Table 3:** Resistance mechanism summary ESBLs, extended spectrum β-lactamases; MDR, multidrug resistance; MBL, metallo-beta-lactamases; XDR, extensively drug-resistant

Category	Factor	Function	Clinical significance
Resistance mechanism	ESBLs	Hydrolyze extended-spectrum cephalosporins and aztreonam	Limits effectiveness of β-lactam antibiotics and promotes MDR
	AmpC β-lactamases	Confer resistance to cephamycins and multiple β-lactam agents	Reduces treatment options and complicates laboratory detection
	Class A carbapenemases	Carbapenem-hydrolyzing enzymes causing broad β-lactam resistance	Associated with extensive MDR
	Class B carbapenemases (MBL)	Zinc-dependent enzymes that hydrolyze most β-lactam antibiotics	Leave very limited therapeutic options and facilitate MDR spread
	Class D carbapenemases	Weakly expressed carbapenemases that may escape routine detection	Can result in XDR phenotypes
	Plasmid-mediated gene transfer	Horizontal transfer of ESBL, AmpC, and carbapenemase genes	Accelerates dissemination of antimicrobial resistance among bacteria

Clinical outcomes

*Proteus* species, particularly *P. mirabilis*, are associated with significant clinical morbidity because of their virulence factors, biofilm-forming ability, and increasing antimicrobial resistance. CAUTIs caused by *Proteus* frequently result in recurrent infections, urinary stone formation, catheter blockage, pyelonephritis, and, in severe cases, urosepsis. Biofilm formation on indwelling medical devices contributes to persistent infections and treatment failure. In wound and burn infections, *Proteus* species are associated with delayed wound healing, prolonged hospitalization, an increased risk of secondary infections, and higher healthcare costs. Gastrointestinal involvement may contribute to chronic intestinal inflammation and disease progression in susceptible individuals. Although uncommon, invasive infections such as bacteremia and infective endocarditis can lead to severe systemic complications, including sepsis, heart valve damage, and increased mortality. The emergence of MDR strains producing ESBLs, AmpC β-lactamases, and carbapenemases has significantly reduced the available treatment options, resulting in prolonged hospital stays, increased antibiotic consumption, higher treatment costs, and poorer patient outcomes. Therefore, early diagnosis, appropriate antimicrobial therapy, effective infection control measures, and continuous surveillance of antimicrobial resistance patterns are essential to reduce the clinical burden associated with *Proteus* infections.

## Conclusions

*Proteus* species remain clinically significant opportunistic pathogens because of their remarkable motility, diverse virulence mechanisms, and increasing antimicrobial resistance. Their ability to swarm, produce urease, form biofilms, and express multiple adhesins and toxins contributes significantly to the development of CAUTIs, wound infections, gastrointestinal disorders, and other clinical conditions. The growing emergence of MDR strains producing ESBLs, AmpC β-lactamases, and carbapenemases has further complicated treatment and infection control practices. Furthermore, the ability of these organisms to acquire and disseminate resistance determinants through plasmids poses a significant public health concern. Accurate laboratory identification, continuous surveillance of antimicrobial resistance patterns, and early detection of resistant phenotypes are therefore essential for effective patient management. A better understanding of the pathogenic mechanisms, virulence factors, and resistance profiles of *Proteus* species will facilitate the development of more effective diagnostic, therapeutic, and preventive strategies against these increasingly challenging infections.
